# Adding muscle power exercises to a strength training program for people with patellofemoral pain: protocol of a randomized controlled trial

**DOI:** 10.1186/s13063-021-05748-x

**Published:** 2021-11-06

**Authors:** Gabriela Souza de Vasconcelos, Guilherme Silva Nunes, Christian John Barton, Raquel Fantinelli Munhoz, Maria Eduarda Chinotti Batista da Silva, Giulia Keppe Pisani, Bruna Calazans Luz, Fábio Viadanna Serrão

**Affiliations:** 1 Physical Therapy Department, Federal University of Sao Carlos (UFSCar), Rodovia Washington Luis Km 235, São Carlos, São Paulo, CEP 13565-905 Brazil; 2 Physiotherapy and Rehabilitation Department, Federal University of Santa Maria (UFSM), Av Roraima 1000, Santa Maria, Rio Grande do Sul CEP 97105-900 Brazil; 3 La Trobe Sport and Exercise Medicine Research Centre, School of Allied Health, La Trobe University, Melbourne, Australia; 4 Department of Surgery, St Vincent’s Hospital, University of Melbourne, Melbourne, Australia

**Keywords:** Knee, Muscle strength, Exercise therapy, Quality of life

## Abstract

**Background:**

Strong evidence supports the proximal combined with quadriceps strengthening for patellofemoral pain (PFP) rehabilitation. However, most reported rehabilitation programs do not follow specific exercise prescription recommendations or do not provide adequate details for replication in clinical practice. Furthermore, people with PFP have power deficits in hip and knee muscles and it remains unknown whether the addition of power exercises would result in superior or more consistent outcomes. Therefore, this study is designed to verify whether the benefits of a rehabilitation program addressing proximal and knee muscles comprising power and strength exercises are greater than those of a program consisting of strength exercises only.

**Method:**

This study will be a randomized controlled trial that will be conducted at university facilities. A minimum of 74 people with PFP between the ages of 18 and 45 years will be included. The experimental group will engage in a 12-week resistance training program focusing on proximal and knee muscles using power and strength exercises. The control group will engage in a 12-week resistance training program focusing on proximal and knee muscles using strength exercises only. Primary outcomes will be pain intensity and physical function; and secondary outcomes will be kinesiophobia, self-reported improvement, quality of life, peak hip and knee torque, and hip and knee rate of force development. The primary outcomes will be evaluated at baseline, and after 6 weeks, 12 weeks, 3 months, 6 months, and 1 year. The secondary outcomes will be evaluated at baseline and immediately after the interventions. Therapists and participants will not be blinded to group allocation.

**Discussion:**

This randomized clinical trial will investigate if adding power exercises to a progressive resistance training may lead to more consistent outcomes for PFP rehabilitation. The study will provide additional knowledge to support rehabilitation programs for people with PFP.

**Trial registration:**

ClinicalTrials.gov NCT 03985254. Registered on 26 August 2019.

**Supplementary Information:**

The online version contains supplementary material available at 10.1186/s13063-021-05748-x.

## Introduction

Patellofemoral pain (PFP) is characterized by retropatellar and/or peripatellar pain that is aggravated during activities that increase patellofemoral joint loading (e.g., squatting and ascending/descending stairs) [1], with an annual prevalence in the general population of around 22.7% [2]. Although PFP was previously considered as a self-limiting condition, recent studies suggest that alterations and symptoms may persist for several years [3–5]. Chronic pain associated with PFP has a negative impact on an individual´s level of physical activity and quality of life, interfering with work, activities of daily living, and physical exercise [5–7].

Previous studies reported that people with PFP have decreased strength in hip [8, 9] and knee muscles [10, 11]. Additionally, other parameters of hip and knee muscle capacity seem to be altered in people with PFP, such as rate of force development (RFD) and power [12–15]. Therefore, exercise therapy addressing these deficits may be important for PFP rehabilitation. Some studies reported the effects of rehabilitation programs for PFP patients focused on proximal muscles combined with quadriceps strengthening [16–23]. Two systematic reviews concluded that the combination of proximal and quadriceps rehabilitation results in greater benefits on pain intensity and physical function compared to isolated quadriceps rehabilitation [24, 25]. Nowadays, the combination of hip and knee muscles exercises presents the best level of evidence for pain reduction and physical function improvement during PFP rehabilitation [26]. Despite improvements in pain and physical function, the effects of resistance training on other characteristics seen in people with PFP such as kinesiophobia, quality of life, and catastrophization are unclear [27–30].

According to the American College of Sports Medicine (ACSM), to achieve sustained improvements in muscle capacity, specific evidence-based guidelines must be followed, such as load progression, number of sets and repetitions, time to rest, weekly frequency, and duration of this training [31, 32]. In a systematic review conducted by Lack et al. [24], the included studies evaluated the effects of resistance training targeting proximal muscles of people with PFP. After further analysis using the ACSM criteria, the authors concluded that three out of 14 studies actually incorporated protocols which could evoke improvements in muscle strength [18, 21, 22]. However, none of the included studies completely accomplished the ACSM guidelines for structural strength gains (more than 8 weeks), as the protocols lasted 4 [18, 21] to 8 weeks [22]. Additionally, according to Lack et al. [24], only one study included power exercises [33], even though the exercise protocol was not clearly described for replication in clinical practice or research. This is a common issue among PFP randomized controlled trials highlighted in the systematic review by Holden et al. [34]. The systematic review included 38 studies and the authors concluded that exercise prescriptions are poorly described, which prevents them from being implemented in clinical practice.

Although hip and knee strength exercises present the best level of evidence for pain reduction and physical function improvement in people with PFP [24–26], little is known in relation to their long-term effects. To date, only the study by Fukuda et al. [21] investigated the long-term effects of exercise (12 months) and the study reported that hip and knee strength exercises result in greater benefits on pain intensity and physical function compared to quadriceps strengthening alone. However, rehabilitation programs with exercises that correct other muscle capacity deficits, such as muscle power, can result in superior or more persistent benefits than those with only strengthening exercises. More recently, Barton et al. [30] demonstrated that a 12-week progressive resistance training program (hip and knee strength and power exercises) is feasible and is associated with improvements in pain, physical function, and muscle capacity (strength and power) in people with PFP. Nonetheless, it is unknown whether the benefits of a proximal combined with quadriceps rehabilitation comprising power and strength exercises are greater than those of a proximal combined with quadriceps rehabilitation consisting of strength exercises only.

To improve the knowledge regarding the effects of muscle power exercises on PFP rehabilitation, the main aim of the study will be to verify if including power exercises to a strength training program addressing proximal and knee muscles provides better outcomes in relation to intensity pain and physical function, compared to a strength training program only, in the short, medium, and long term in people with PFP. Furthermore, the aim will be to verify whether the proximal combined with quadriceps rehabilitation comprising power and strength exercises will result in superior benefits on kinesiophobia, self-reported improvement, quality of life, and muscle capacity (increased peak hip abduction and extension torque, and peak knee extension torque; increased hip abduction and extension RFD, and knee extension RFD). The hypothesis of the study is that people with PFP undergoing the proximal combined with quadriceps rehabilitation comprising power and strength exercises will improve considerably concerning the evaluated outcomes when compared to people undergoing the proximal combined with quadriceps rehabilitation composed by strength exercises only.

## Methods

### Trial design

**Fig. 1:**
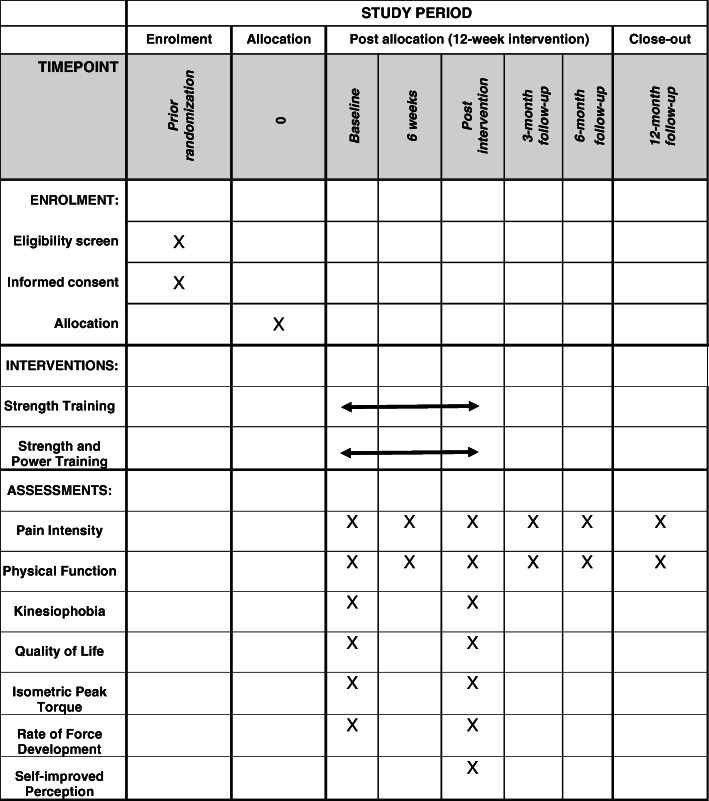
Participant timeline: schedule of enrolment, interventions, and assessments

This is a randomized controlled trial. The study will be developed in six stages, according to Fig. 1. The baseline assessment will consist of evaluating anthropometric and demographic data, in addition to pain intensity, physical function, kinesiophobia, quality of life, and muscle capacity. After 1 week, the intervention phase will be initiated, which will consist of two different intervention protocols for 12 weeks, according to the group allocation. Six weeks after starting the intervention, the intensity of pain, physical function, kinesiophobia, and quality of life will be assessed. At the end of the intervention protocols, the participants will be evaluated for pain intensity, physical function, kinesiophobia, quality of life, muscle capacity, and self-reported improvement. After 3 months, 6 months, and 1 year, participants will be re-evaluated for pain intensity and physical function through online questionnaires. The lower limb of the affected side will be assessed. In cases of bilateral symptoms, the more painful lower limb will be assessed [12].

The study will be conducted at the Laboratory of Evaluation and Intervention in Orthopedics and Traumatology (LAIOT) at the Federal University of São Carlos (UFSCar), São Carlos, São Paulo, Brazil, and will follow the CONSORT guideline [35] and the TIDieR checklist to describe the interventions [36]. The present protocol was reported according to the SPIRIT guideline [37].

### Ethics

The research was approved by the University Research Ethics Committee (CAAE: 12417019.8.0000.5504). Each participant will receive explanations regarding research objectives, anonymity of their data, and freedom to participate. Participants will sign an Informed Consent Term (ICT). This study will respect the ethical precepts of Resolution CNS 510/2016 and be performed according to the Declaration of Helsinki. It was registered in ClinicalTrials.gov, on 26 August 2019, under number ID: NCT 03985254.

### Participants and setting

Potential participants will be recruited from the community, gyms, or social media, and a minimum of 74 participants will be included in the study. A researcher will perform a preliminary screening of the eligibility criteria and will explain the study procedures during a telephone call. If the potential participants likely fulfill the inclusion criteria and declare their agreement to participate, the diagnosis of PFP will be confirmed by a physiotherapist through a physical examination [38]. This physiotherapist (BCL) will explain the study procedures and will obtain written consent from patients willing to participate in the trial.

To be included, the participants should meet the following inclusion criteria: (i) men and women affected by PFP (unilateral or bilateral) aged between 18 and 45 years; (ii) insidious onset of symptoms unrelated to a traumatic event; (iii) presenting retropatellar or peripatellar pain (3/10 points according to the visual analog scale—VAS) in at least two of the following functional activities: stair negotiation, running, kneeling, squatting, sitting for long periods, or jumping; and (iv) presence of pain for at least 2 months [17]. The exclusion criteria will be history of surgery to the knees; history of injury or pain in the hip; patellar instability; pain on palpation of the patellar tendon, iliotibial band, Hoffa fat, pes anserinus tendons or knee joint line; signs or symptoms of meniscal or ligamentous knee injuries; presence of Osgood-Schlatter or Sinding-Larsen-Johansson syndrome; and any vestibular, neurological, or musculoskeletal alterations that interfere with or contraindicate the measurement procedures of this study [22, 39]. After randomization, the criterion for dropping out will be the participants not attending the assessments (6 weeks, post intervention and follow-ups).

### Sample size

The sample size of this study was calculated considering a statistical power of 80%, alpha of 5%, and an estimated 15% of sample losses. In order to detect a 2-point difference in pain NRS [40] with a standard deviation of 2.8 points [41], a sample size calculation indicated 37 participants in each group.

### Randomization and allocation

All participants who give consent for participation and who fulfill the inclusion criteria will be randomized to the Strength Training Group (STG) or to the Power and Strength Training Group (PSTG) with a 1:1 allocation ratio. Randomization will be performed using consecutively numbered, sealed and opaque envelopes previously prepared and randomly assigned by a random number generator program (www.randomization.com). Allocation concealment will be ensured, since the randomization code will only be released after the participant has been included in the clinical trial. A researcher not involved in the assessment process will perform the randomization and participant allocation to the groups by opening the envelopes after the baseline evaluation. The physiotherapist, responsible for the intervention, will open the envelope and, will find the treatment condition to be conducted in this participant. The researcher responsible for assessments is not allowed to receive information about the group allocation. Thus, randomization will be conducted without any influence of the physiotherapists and researchers responsible for assessments and interventions.

### Interventions

Participants from both groups will perform the supervised training program three times per week for 12 weeks, with at least 24 h of interval between intervention sessions. No instruction for home exercise will be given. Participants will be instructed to maintain their physical and sports activities.

The duration of each session for the STG will be around 60 min and for the SPTG around 75 min. All sessions will be supervised by an experienced physiotherapist (> 5 years). The description of the exercises has been made simple and clear; therefore, physiotherapists will need minimal training to apply the exercises (Additional file 1 – supplementary material).

The STG and SPTG programs will be based on the training principles recommended by the American College of Sport Medicine (ACSM) [32]. The exercises were chosen based on a study that successfully applied the protocol to people with PFP [30] and other strength training studies [22, 42–45].

To perform the exercises, ankle weights, free weights and/or dumbbells, and elastic bands will be used. The load to be used in the exercises of both groups will be determined based on the 10RM (repetition maximum) test to estimate the 1RM—the 10RM load indicates approximately 75% of the 1RM load [46]. As the participants have PFP, the 1RM test could exacerbate the symptoms. For exercises using an elastic band, the 1RM test will be performed. The 1RM will be considered the highest elastic band (considering the color of the elastic), in which the participant can perform a single repetition [22].

Regarding pain management during exercise, a level equal to or lower than three points in VAS will be accepted, and if any participant presents pain above this level, the exercise will be modified according to the variations that are also in the exercise protocol (Additional file 1). In addition to pain management, the presence of muscle failure will also be monitored. Exercise will be interrupted if muscle failure occurs, which will be considered as the participant's inability to move a specific load beyond a critical joint angle [47] or as the inability to complete a repetition in the stipulated range of motion due to fatigue [48].

As an adherence strategy to encourage participants attending training sessions, both groups will receive face-to-face education sessions about possible causes and consequences of PFP, pain management, importance of physical activity for the treatment, and other questions that may arise during the intervention period. In addition, telephone calls will be made to remind participants about the sessions. The intention is that the participants feel part of the treatment, understand how the intervention can help them, and, thus, attend the intervention sessions [49].

### Strength Training Group (STG)

**Fig. 2:**
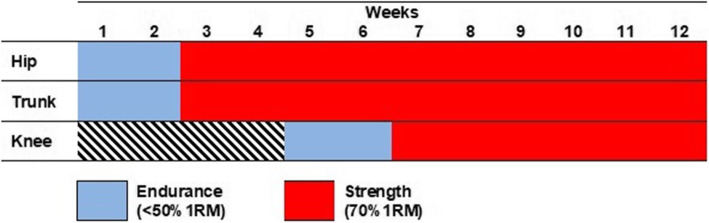
Strength Training Group (STG) schedule

The strength training program will consist of applying resistance and progressive exercises for strength gain. Initially, the goal will be to develop neuromotor control and endurance (load < 50% 1RM), and in subsequent weeks, the goal will be to develop muscle strength (load > 70% 1RM) (Fig. 2). The protocol will initially focus on hip and trunk muscles, and after 4 weeks of training, knee muscle exercises (weight-bearing or non-weightbearing) will be included.

At each training session, at least five exercises (out of a total of 15 exercises) will be chosen and applied by the therapist. From these five exercises, one will be for hip extensors, one for hip abductors, one for knee extensors, and two for the trunk. Additional file 1 provides a complete description of all exercises that may be applied to participants. According to the evolution of the participants, regarding pain and ease of execution, the therapist will perform the progression of the loads and even the substitution of one exercise for a more complex one as long as it is one of the program exercises.

### Power and Strength Training Group (PSTG)

**Fig. 3:**
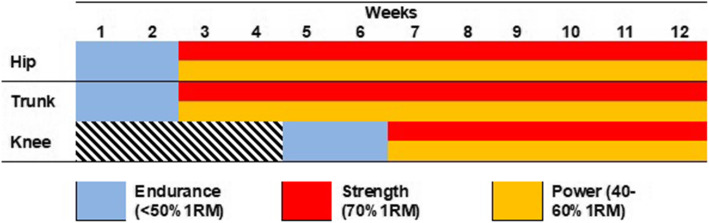
Power and Strength Training Group (PSTG) schedule

Participants assigned to this group will perform the same exercise program as the STG and power training exercises (fourth column of Additional file 1). Initially, the focus will be on neuromotor control and resistance development (load < 50% 1RM). Afterwards, the goal will be to develop strength (load> 70% 1RM) and muscle power (load between 40 and 60% 1RM) (Fig. 3).

### Outcome measures

Primary outcomes will be pain intensity and physical function, and secondary outcomes will be kinesiophobia, self-reported improvement, quality of life, peak hip and knee torque, and hip and knee rate of force development. The primary outcomes will be evaluated at baseline, and after 6 weeks, 12 weeks, 3 months, 6 months, and 1 year. The secondary outcomes will be evaluated at baseline and immediately after the interventions.

### Pain intensity

Pain intensity will be measured using the Numeric Rating Scale (NRS-11), where 0 represents “no pain” and 10 represents “the worst pain possible.” Participants will indicate their usual pain and worst pain during the past week [40].

### Physical function

The Anterior Knee Pain Scale (AKPS), translated and validated into Portuguese [50], will be used to assess subjective symptoms such as anterior knee pain and functional limitations related to PFP. The items evaluated in the questionnaire are patellar subluxation, limp, pain, walking, climbing stairs, and sitting for a long time with bent knees. It has a score from 0 to 100 points, where 100 means no pain and/or functional limitations and 0 means constant pain and various functional limitations [51]. The AKPS is a reliable and valid instrument for assessing function in individuals with PFP [40].

### Kinesiophobia

The evaluation of kinesiophobia will be performed by the translated and validated Portuguese version of the Tampa Scale for the Kinesiophobia questionnaire [52], which consists of 17 items that assess fear of movement, injury or recurrence of injury [53]. This questionnaire is a four-point Likert scale, where the sum of the answers can vary from 17 to 68, and scores higher than 37 indicate the presence of kinesiophobia [54].

### Quality of Life

Participants will be assessed using the specific subscale for quality-of-life assessment of Knee Injury and Osteoarthritis Outcome Score (KOOS). This subscale comprises four questions and each one will be scored from 0 to 4, where 0 represents extreme knee problems and 4 that there are no knee problems. The sum of these questions will be used for further analysis [55, 56].

### Muscle capacity

Isometric torque and RFD of knee extensors, hip extensors, and abductors will be evaluated using an isokinetic dynamometer (Biodex Multi Joint System 3, Biodex Medical Systems Inc., New York, USA), with a sampling frequency of 100 Hz.

- Knee extensors: the participants will be placed in a sitting position with hips at 85° of flexion and neutral position for transversal and frontal planes, and the knee of the assessed limb at 60° of flexion [13]. The resistance pad will be fixed with a velcro strip just above the lateral malleolus and the rotation axis of the dynamometer will be aligned with the lateral femoral epicondyle.
- Hip extensors: the participants will be in a prone position on the testing table of the dynamometer and their legs will be off the table. The assessed lower limb will be placed with the hip at 30° of flexion, and the participants should keep the knee at 90° of flexion and avoid hip rotations during the test. The resistance pad will be fixed with a velcro strip just above the popliteal fossa, and the rotation axis of the dynamometer will be aligned with the center of the hip joint in the sagittal plane near the greater trochanter of the femur [12, 57].
- Hip abductors: the participants will be placed in a side-lying position, whereby the top leg will be the assessed side. The assessed hip will be in neutral position for all three planes, the knee will also be in neutral position, and the participants will be instructed to keep their toes pointed forward and not to bend their knee during the test [58]. The resistance pad will be fixed with a velcro strip 5 cm above the upper edge of the patella and the rotation axis of the dynamometer will be aligned to a point representing the intersection of two lines: one line directed inferiorly from the posterior superior iliac spine towards the knee, and the other line oriented medially and posteriorly to the greater trochanter of the femur toward the midline of the body.

For the whole assessment, the participants should perform the contraction as powerfully and as quickly as possible and maintain the contraction for 5 s. For each assessed muscle group, three maximal contractions will be collected with a 1-min interval between them. The participants will be verbally encouraged to achieve maximum power throughout all the contractions. Previously to the data collection, the participants will be familiarized with the tests by performing two submaximal contractions and one maximal contraction. A 3-min interval will be adopted between familiarization and data collection. Isometric torque measures will be normalized by body mass (Nm/kg × 100), and the highest value of the three repetitions will be used for statistical analysis for each muscle group [12].

A test-retest reliability study will be conducted to verify intra-examiner reliability of measurements by evaluating 10 participants in two moments, with an interval of 3 to 7 days.

To calculate the RFD, the highest value of the three repetitions for each muscle group will be used [12]. The normalized torque data will be exported and processed in the Matlab Software (Mathworks, Natick, Massachusetts, USA, version 2008b). RFD will be calculated using the slope of the torque/time curve. The slope will be obtained by dividing the variation of the normalized torque (Nm/Kg X 100, represented as %) by the time variation (ms) from the start of the contraction until 30% and 90% of the maximal isometric torque [59, 60]. The beginning of the contraction will be defined as the moment when the isometric torque exceeds 2% of the peak torque [61]. Thus, higher RFD values indicate an increased ability to generate force rapidly [62].

### Self-reported improvement

The self-reported improvement with reference to the start of the study will be quantified using the Global Rating of Change (GROC). This tool is a 15-point Likert-type scale that measures a patient's perception of a change in knee pain following a specific treatment [63]. The scale ranges from − 7 (a very great deal worse) to + 7 (a very great deal better), with 0 (zero) indicating no change. Changes of 4 points or more on this scale have been previously considered as clinically important in patients with knee pain [64].

### Blinding

The researcher in charge of the evaluations and the data processing will be blinded to the group allocation. The participants and the therapist will not be blinded to the group allocation due to the differences between our interventions which are easily detectable.

### Minimizing missing data

In order to avoid and minimize missing data, as an adherence strategy to encourage participants to attend training sessions, both groups will receive face-to-face education sessions about possible causes and consequences of PFP, pain management, importance of physical activity for the treatment, and other questions that may arise during the intervention period. In addition, telephone contacts will be made to remind participants about the sessions and the evaluations. Regarding the 3 month-, 6 month-, and 1-year follow-ups, the researcher will contact the participant by telephone and send them the online questionnaire.

### Data management

Data are being collected at the Laboratory of Evaluation and Intervention in Orthopedics and Traumatology (LAIOT) at the Federal University of São Carlos (UFSCar). Data will be stored at the Physical Therapy Department at UFSCar with a password-protected computer file, to which only researchers will have access. The main researcher will have a backup copy of all the information.

The study results will be released in conferences, such as scientific conferences, internationally and nationally, and through articles published in peer-reviewed journals. This research is a part of a PhD thesis, and the publishing rights are owned by the authors. The results will be frequently presented to the supervising professor (FVS).

### Data monitoring

Researchers will monitor participants throughout the study development (assessment and interventions). The supervising professor (FVS), who will be blind to the group allocations, will monitor any adverse effects and perform database management and statistical analyses.

Adverse events during the study procedures, either during assessments, interventions, or follow-up, will be registered and reported. Adverse events will be considered any symptom or disease that is related or not to the evaluations and the intervention.

The University Graduate Program will supervise the integrity of the data, and the responsible Internal Data Monitoring Committee will have access to the patient allocation, while the whole analysis will be confidential. The supervising professor (FVS) will ensure that the University Graduate Program and Internal Data Monitoring Committee will be provided with access to source data/documents, ensuring the confidentiality of the participants.

### Harm

Participant data will be carefully accessed, and all harm and complications of the treatment will be reported together with the other results of this trial, if any.

### Auditing

The University Graduate Program will supervise the integrity of the data. Moreover, the results will be frequently presented to the supervising professor (FVS), who will be blind to the group allocations, and will verify if data are accurate, complete, and verifiable and that the conduct of the study complies with the currently approved protocol.

### Statistical analysis

The intention-to-treat approach will be applied for all analyses [65]. The differences from baseline will be used in the analysis. The effects of intervention on the outcome measures will be assessed by analysis of variance (ANOVA). For pain and physical function (primary outcomes), a 2-by-6 analysis of variance will be used with the groups (STG and PSTG) as the independent factor and time (baseline, 6-week, post intervention, 3-month follow-up, 6-month follow-up, and 12-month follow-up) as the repeated factor. For kinesiophobia (secondary outcome), a 2-by-3 analysis of variance (2 groups and 3 time points) will be used. For quality of life and muscle capacity (hip and knee torque; hip and knee RFD) (secondary outcomes), a 2-by-2 analysis of variance (2 groups and 2 time points) will be used. For GROC outcome (secondary outcomes), the reference criterion of treatment success will be a score of + 6 (much improved) or higher and these data will be presented as a percentage. Moreover, chi-square tests will be performed to compare the percentage of patients who perceived much improvement in each group based on the GROC scale. Separate models will be used for each outcome measure. For significant group-by-time interactions, planned pairwise comparisons with post hoc Bonferroni will be used to determine differences. The mean difference and 95% CI will be also calculated for each comparison. In cases of missing data, the multiple imputation method will be adopted to impute missing values [66], and per-protocol analysis will be also performed. The significance level will be 0.05. All the statistical analysis will be performed using a Statistical Package for the Social Sciences software program (version 20.0, SPSS, Inc., Chicago, IL, USA), and the researcher who will perform the analysis will be blinded to the group allocation. Interim analyses will not be performed. No additional analyses will be performed in the trial.

## Discussion

Knee pain is the second most prevalent condition, in which PFP is considered one of the most common forms of knee pain [67]. People with PFP have decreased hip [8, 9] and knee muscle strength [10, 11], and, as a result, many studies have focused on improving these deficits [16–23]. According to the latest PFP consensus [26], the proximal combined with quadriceps strengthening is the physiotherapeutic procedure that has the best evidence for pain reduction and function improvement in PFP patients.

Moreover, people with PFP present other proximal and knee muscle capacity deficits, such as rate of force development (RFD) and power [12–15]. Nunes et al. [12, 13] reported lower hip abductor and extensor and knee extensor rate of force development (RFD) during maximal isometric contraction in women with PFP compared to women without PFP. Similarly, according to the study by Ferreira et al. [15], women with PFP have lower hip abductor and knee extensor RFD during isometric, concentric, and eccentric contractions compared to women without PFP. Finally, using a linear position transducer, it was found that people with PFP had deficits of 31% and 29% in hip abductor and extensor power, respectively [14].

Although these recent studies have shown that patients with PFP have hip and knee muscle power deficits [12–15], little is known in relation to power training effects in these people. Until the present moment, only two studies included power exercises for the treatment of these patients [30, 33]. Nevertheless, Tyler et al. [33] did not adequately describe which power exercises were applied, and Barton et al. [30] did not have a comparator group (isolated muscle strength protocol) to clarify whether adding power exercises to a strength training protocol results in greater benefits than muscle strength training alone.

In addition to these problems, others are identified in previously published studies with interventions for PFP. For example, the vast majority of them did not follow ACSM recommendations aiming to gain muscle strength (load progression, number of sets and repetitions, time to rest, weekly frequency and duration of this training) [24]. Another problem, presented in the systematic review by Holden et al. [34]^,^ is that they were not clearly described (exercise prescriptions), making it difficult to apply them in clinical practice. And these problems can interfere with long-term results.

Therefore, this study will be the first clinical trial to compare the effects of adding power exercises to strength training with an isolated strength training program. Moreover, it will also analyze if these effects will be longer lasting than those of isolated strength training. The protocols will follow ACSM recommendations and will be described in detail to facilitate applications in clinical practice, and that could favor long-term benefits.

In relation to benefits, hip and knee strength training alone is able to improve pain and physical function in people with PFP [24–26], but only one study has evaluated long-term effects [21]. Adding power exercises to strength training can promote more consistent results in people with PFP. Furthermore, these results may be longer lasting than those of strength training alone (12 months).

Depending on the results of our study, we aim to change physiotherapist clinical practice by targeting hip and knee power exercises as a part of routine treatment for people with PFP. Usually, treatment for people with PFP includes hip and knee strength exercises and passive adjuncts (orthoses and insoles) and little or no attention to power exercises. In addition, because it follows the recommendations of the ACSM and exercises are described in detail (according to TIDieR), the protocol can be replicated in clinical practice and/or in scientific research.

## Trial status

This trial is currently recruiting patients, the first patient was included on 12 August 2019, and this is the original version. To date, we have enrolled 37 study participants who have completed treatment, assessments, and follow-ups. We predict that recruitment will be completed in December 2021. Although existing data are being entered, no analysis has been performed yet.

## Supplementary Information

**Additional file 1:** Supplementary Material.

## Data Availability

The dataset(s) supporting the conclusions of this article are available upon request from the authors.
