# Supplementary material for: Adding muscle power exercises to a strength training program for people with patellofemoral pain: protocol of a randomized controlled trial
Source: Trials. 2021 Nov 6;22:777. doi: 10.1186/s13063-021-05748-x (PMC8572497; doi:10.1186/s13063-021-05748-x)
Supplement: Supplementary file 1 — Additional file 1: Supplementary Material. [file 13063_2021_5748_MOESM1_ESM.pdf]

Appendix A - Exercise protocol

Hip Extensors

Exercise	Resistance Training	Strength Training	Power Training
**Bridge**<br>Description: Participant in supine position with the back on the floor, arms crossed in front of the chest, hips and knees at 45 and 90° of flexion, respectively. The participant lifts the buttocks off the floor until the hips reach the neutral position (concentric phase) and then returns to the starting position (eccentric phase).<br><br>Starting position<br>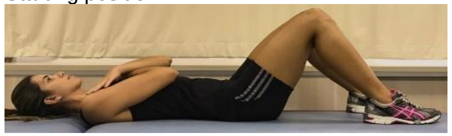<br>Execution<br>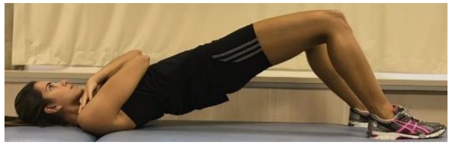	Initial load: 50% 1RM<br><br>3 sets x 20 reps<br><br>1-minute rest between sets<br>1 second rest between repetitions<br><br>Time under tension/reps: 5 seconds<br>Concentric phase: 2 seconds<br>Isometric Phase: 1 second<br>Eccentric phase: 2 seconds<br><br>Regression: decrease load (45% 1RM) or the number of repetitions (up to 15)<br><br>Progression: increase load (2-10% 1 RM) or the number of repetitions (up to 25)	Initial load: 70% 1RM<br><br>3 sets x 12 reps<br><br>2-3 minutes of rest between sets<br>1 second rest between repetitions<br><br>Time under tension/reps: 5 seconds<br>Concentric phase: 2 seconds<br>Isometric Phase: 1 second<br>Eccentric phase: 2 seconds<br><br>Regression: decrease load (up to 60% 1 RM) or the number of repetitions (up to 8)<br><br>Progression: increase load (2-10% 1RM) and/or perform with unilateral support	Initial load: 40 - 60% 1RM<br><br>3 sets x 6 reps<br><br>2-3 minutes of rest between sets<br><br>Time under tension/reps: <2 seconds<br>Concentric phase: <1 second<br>Eccentric phase: 1 second<br><br>Regression: decrease load (up to 30% 1RM)<br><br>Progression: increase the speed of the concentric phase
**Prone Hip Extension**<br>Description: Participant in prone position, with the knee on the side that will perform the exercise flexed at 90° and the ankle weight fixed to the distal third of the thigh. The participant performs the hip extension (concentric phase) and then returns to the initial position (eccentric phase). This is performed on both sides.	Initial load: 50% 1RM<br><br>3 sets x 20 reps<br><br>1-minute rest between sets<br>1 second rest between repetitions<br><br>Time under tension/reps: 4 seconds<br>Concentric phase: 2 seconds<br>Eccentric phase: 2 seconds	Initial load: 70% 1RM<br><br>3 sets x 12 reps<br><br>2-3 minutes of rest between sets<br>1 second rest between repetitions<br><br>Time under tension/reps: 4 seconds<br>Concentric phase: 2 seconds<br>Eccentric phase: 2 seconds	Initial load: 40 - 60% 1RM<br><br>3 sets x 6 reps<br><br>2-3 minutes of rest between sets<br><br>Time under tension/reps: <2 seconds<br>Concentric phase: <1 second<br>Eccentric phase: 1 second<br><br>Regression: decrease load (up to 30% 1RM)

Starting position<br>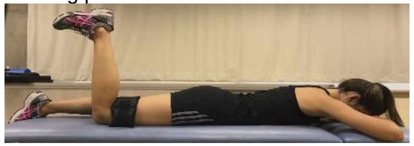<br>Execution<br>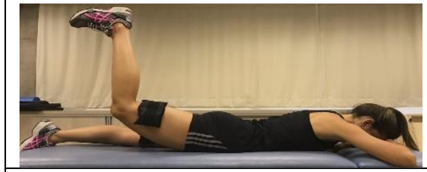	Regression: decrease load (45% 1RM) or the number of repetitions (up to 15)<br><br>Progression: increase load (2-10% 1 RM) or the number of repetitions (up to 25)	Regression: decrease load (60% 1 RM) or the number of repetitions (up to 8)<br><br>Progression: increase load (2-10% 1RM)	Progression: increase the speed of the concentric phase
**Hip extension in quadruped position**<br>Description: Participant starts the exercise in quadruped position and with and the ankle weight fixed to the distal third of the leg. The participant performs the hip extension up to 0°, keeping the knee in 90° of flexion (concentric phase), and returns to the initial position (eccentric phase). This is performed on both sides.<br><br>Starting position<br>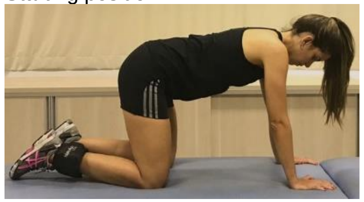<br>Execution<br>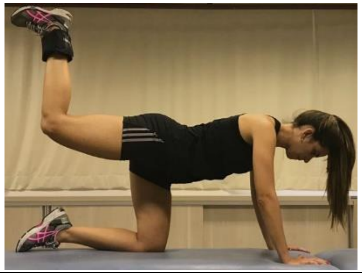	Initial load: 50% 1RM<br><br>3 sets x 20 reps<br><br>1-minute rest between sets<br>1 second rest between repetitions<br><br>Time under tension/reps: 4 seconds<br>Concentric phase: 2 seconds<br>Eccentric phase: 2 seconds<br><br>Regression: decrease load (45% 1RM) or the number of repetitions (up to 15)<br><br>Progression: increase load (2-10% 1 RM) or the number of repetitions (up to 25)	Initial load: 70% 1RM<br><br>3 sets x 12 reps<br><br>2-3 minutes of rest between sets<br>1 second rest between repetitions<br><br>Time under tension/reps: 4 seconds<br>Concentric phase: 2 seconds<br>Eccentric phase: 2 seconds<br><br>Regression: decrease load (60% 1 RM) or the number of repetitions (up to 8)<br><br>Progression: increase load (2-10% 1RM)	Initial load: 40 - 60% 1RM<br><br>3 sets X 6 reps<br><br>2-3 minutes of rest between sets<br><br>Time under tension/reps: <2 seconds<br>Concentric phase: <1 second<br>Eccentric phase: 1 second<br><br>Regression: decrease load (up to 30% 1RM)<br><br>Progression: increase the speed of the concentric phase
**Standing Hip Extension**	Initial load: 2 levels of elastic resistance less than 1RM	Initial load: 1 elastic resistance level less than 1RM	Initial load: 1 elastic resistance level less than 1RM

Description: The participant standing with the hip that will perform the exercise in 30° of flexion. The elastic band will be fixed on the participant's ankle. Then, the participant extends the hips until reaching 20° of extension (concentric phase) and returns to the initial position (eccentric phase). This is performed on both sides. The participant can hold a stick to stabilize the upper body.<br><br>Starting position Execution<br>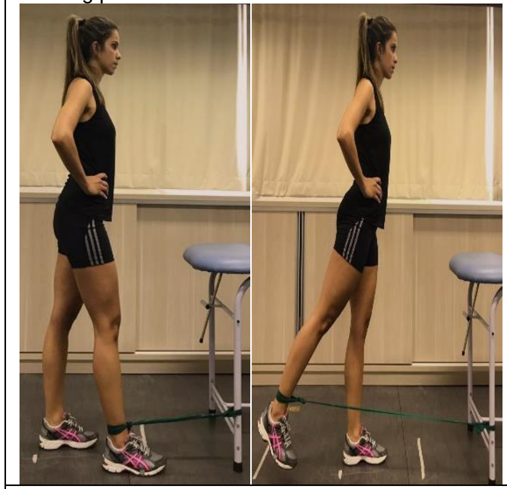	3 sets x 20 reps<br><br>1-minute rest between sets<br>1 second rest between repetitions<br><br>Time under tension/reps: 4 seconds<br>Concentric phase: 2 seconds<br>Eccentric phase: 2 seconds<br><br>Regression: decrease load (1 level of elastic resistance) or the number of repetitions (up to 15)<br><br>Progression: increase load (1 level of elastic resistance) or the number of repetitions (up to 25)	3 sets x 12 reps<br><br>2-3 minutes of rest between sets<br>1 second rest between repetitions<br><br>Time under tension/reps: 4 seconds<br>Concentric phase: 2 seconds<br>Eccentric phase: 2 seconds<br><br>Regression: decrease load (1 level of elastic resistance) or the number of repetitions (up to 8)<br><br>Progression: increase load (1 level of elastic resistance)	3 sets x 6 reps<br><br>2-3 minutes of rest between sets<br><br>Time under tension/reps: <2 seconds<br>Concentric phase: <1 second<br>Eccentric phase: 1 second<br><br>Regression: decrease load (1 level of elastic resistance)<br><br>Progression: increase the speed of the concentric phase
**Deadlift**<br>Description: Participant starts standing up, holding dumbbells or barbell bars. Participant performs trunk and hip flexion on the side that is in support and hip extension on the opposite side (eccentric phase - support side). And then he/she returns to the starting position (concentric phase - support side). This is performed on both sides.	It is suggested that this exercise be performed in the final phase of the training program, in which the participants will have more strength and control over the movements performed.	Initial load: 70% 1RM<br><br>3 sets x 12 reps<br><br>2-3 minutes of rest between sets<br>1 second rest between repetitions<br><br>Time under tension/reps: 4 seconds<br>Eccentric phase: 2 seconds<br>Concentric phase: 2 seconds	This exercise will not be performed for power gain.

Starting position Execution 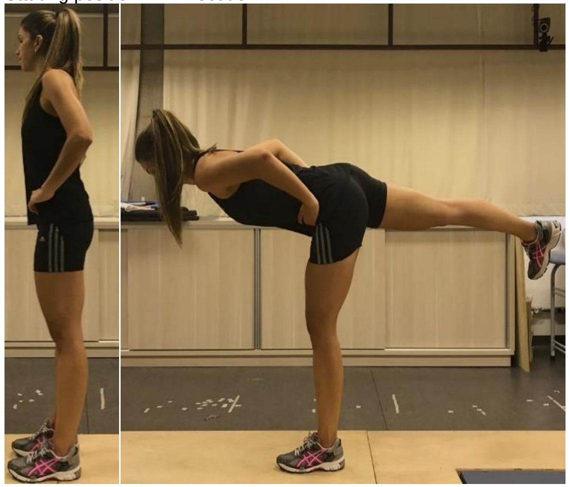		Regression: decrease load (60% 1 RM) or the number of repetitions (up to 8)<br><br>Progression: increase load (2-10%)	

Hip Abductors

Exercise	Resistance Training	Strength Training	Power Training
**Side leg lifts**<br>Description: Participant lying on side, with the ankle weight fixed to the distal third of the leg. The side that will perform the exercise remains in a neutral position and the side that will be below is with approximately 90° of hip and knee flexion, approximately. The participant abducts the hip to the angle where there is no compensation with the pelvic girdle and/or lumbar spine (concentric phase) and returns to the initial position (eccentric phase). This is performed on both sides.<br><br>Starting position<br>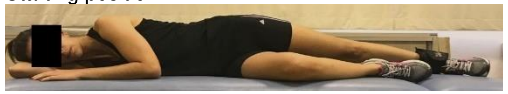<br>Execution<br>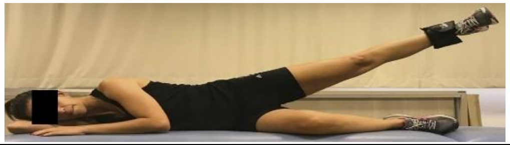	Initial load: 50% 1RM<br><br>3 sets x 20 reps<br><br>1-minute rest between sets<br>1 second rest between repetitions<br><br>Time under tension/reps: 4 seconds<br>Concentric phase: 2 seconds<br>Eccentric phase: 2 seconds<br><br>Regression: decrease load (45% 1RM) or the number of repetitions (up to 15)<br><br>Progression: increase load (2-10% 1 RM) or the number of repetitions (up to 25)	Initial load: 70% 1RM<br><br>3 sets x 12 reps<br><br>2-3 minutes of rest between sets<br>1 second rest between repetitions<br><br>Time under tension/reps: 4 seconds<br>Concentric phase: 2 seconds<br>Eccentric phase: 2 seconds<br><br>Regression: decrease load (60% 1 RM) or the number of repetitions (up to 8)<br><br>Progression: increase load (2-10%)	Initial load: 40 - 60% 1RM<br><br>3 sets x 6 reps<br><br>2-3 minutes of rest between sets<br><br>Time under tension / reps: <2 seconds<br>Concentric phase: <1 second<br>Eccentric phase: 1 second<br><br>Regression: decrease load (up to 30% 1RM)<br><br>Progression: increase the speed of the concentric phase
**Standing hip abduction**<br>Description: The participant is standing with the hips that will perform the exercise in a neutral position. The elastic band will be fixed on the participant's ankle. Then, the participant abducts the hip to the angle where there is no compensation with the pelvic girdle and/or lumbar spine (concentric phase) and returns to the initial position (eccentric phase). This is performed on both sides. The participant can hold a stick to stabilize the upper body.	Initial load: 2 levels of elastic resistance less than 1RM<br><br>3 sets x 20 reps<br><br>1-minute rest between sets<br>1 second rest between repetitions<br><br>Time under tension/reps: 4 seconds<br>Concentric phase: 2 seconds<br>Eccentric phase: 2 seconds	Initial load: 1 elastic resistance level less than 1RM<br><br>3 sets x 12 reps<br><br>2-3 minutes of rest between sets<br>1 second rest between repetitions<br><br>Time under tension/reps: 4 seconds<br>Concentric phase: 2 seconds<br>Eccentric phase: 2 seconds	Initial load: 1 elastic resistance level less than 1RM<br><br>3 sets x 6 reps<br><br>2-3 minutes of rest between sets<br><br>Time under tension/reps: <2 seconds<br>Concentric phase: <1 second<br>Eccentric phase: 1 second

Starting position Execution<br>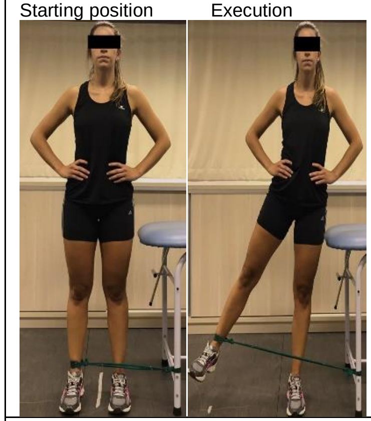	Regression: decrease load (1 level of elastic resistance) or the number of repetitions (up to 15)<br><br>Progression: increase load (1 level of elastic resistance) or the number of repetitions (up to 25)	Regression: decrease load (1 level of elastic resistance) or the number of repetitions (up to 8)<br><br>Progression: increase load (1 level of elastic resistance)	Regression: decrease load (1 level of elastic resistance)<br><br>Progression: increase the speed of the concentric phase
**Pelvic Drop**<br>Description: The participant is standing in single limb support, the knee straight and the foot positioned close to the edge of a step. The contralateral leg is positioned over the floor adjacent to the step (ankle weights are fixed on the distal third of this leg). The participant alternately raises and lowers the limb off the edge of the step (by raising and lowering the pelvis). This is performed on both sides.<br><br>Starting position Execution<br>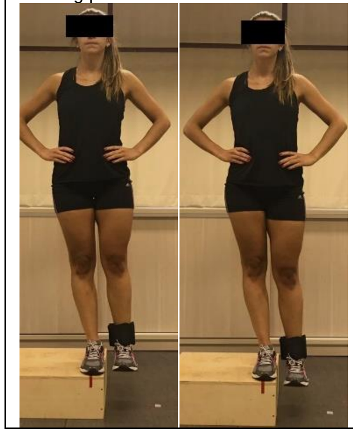	Initial load: 50% 1RM<br><br>3 sets x 20 reps<br><br>1-minute rest between sets<br>1 second rest between repetitions<br><br>Time under tension/reps: 4 seconds<br>Eccentric phase: 2 seconds<br>Concentric phase: 2 seconds<br><br>Regression: decrease load (45% 1RM) or the number of repetitions (up to 15)<br><br>Progression: increase load (2-10% 1 RM) or the number of repetitions (up to 25)	Initial load: 70% 1RM<br><br>3 sets x 12 reps<br><br>2-3 minutes of rest between sets<br>1 second rest between repetitions<br><br>Time under tension/reps: 4 seconds<br>Eccentric phase: 2 seconds<br>Concentric phase: 2 seconds<br><br>Regression: decrease load (60% 1 RM) or the number of repetitions (up to 8)<br><br>Progression: increase load (2-10%)	Initial load: 40 - 60% 1RM<br><br>3 sets x 6 reps<br><br>2-3 minutes of rest between sets<br><br>Time under tension/reps: <2 seconds<br>Eccentric phase: <1 second<br>Concentric phase: 1 second<br><br>Regression: decrease load (up to 30% 1RM)<br><br>Progression: increase the speed of the concentric phase

Knee Extensors

Exercise	Resistance Training	Strength Training	Power Training
**Knee extension with elastic band**<br>Description: Participant starts sitting with knees flexed at 90° and the elastic band is fixed to the participant's ankle. Then, the participant performs the knee extension up to 45° of flexion (concentric phase) and returns to the initial position (eccentric phase).<br>Starting position 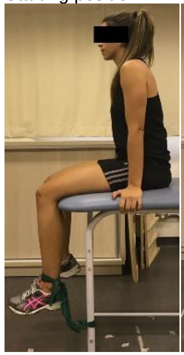 Execution 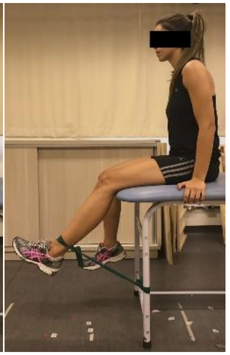	Initial load: 2 levels of elastic resistance less than 1RM<br><br>3 sets x 20 reps<br><br>1-minute rest between sets<br>1 second rest between repetitions<br><br>Time under tension/reps: 4 seconds<br>Concentric phase: 2 seconds<br>Eccentric phase: 2 seconds<br><br>Regression: decrease load (1 level of elastic resistance) or the number of repetitions (up to 15)<br><br>Progression: increase load (1 level of elastic resistance) or the number of repetitions (up to 25)	Initial load: 1 elastic resistance level less than 1RM<br><br>3 sets x 12 reps<br><br>2-3 minutes of rest between sets<br>1 second rest between repetitions<br><br>Time under tension/reps: 4 seconds<br>Concentric phase: 2 seconds<br>Eccentric phase: 2 seconds<br><br>Regression: decrease load (1 level of elastic resistance) or the number of repetitions (up to 8)<br><br>Progression: increase load (1 level of elastic resistance)	Initial load: 1 elastic resistance level less than 1RM<br><br>3 sets x 6 reps<br><br>2-3 minutes of rest between sets<br><br>Time under tension/reps: 4 seconds<br>Concentric phase: <1 second<br>Eccentric phase: 1 second<br><br>Regression: decrease load (1 level of elastic resistance)<br><br>Progression: increase the speed of the concentric phase
**Two Legged Squat**<br>Description: Participant starts standing with feet shoulder-width apart, holding dumbbells. The participant performs the squat at about 90° of flexion of the hips and knees, until touching a bench positioned behind the participant (eccentric phase) and then returns to the starting position (concentric phase).	Initial load: 50% 1RM<br><br>3 sets x 20 reps<br><br>1-minute rest between sets<br>1 second rest between repetitions<br><br>Time under tension/reps: 4 seconds<br>Eccentric phase: 2 seconds<br>Concentric phase: 2 seconds<br><br>Regression: decrease load (45% 1RM) or the number of repetitions (up to 15) or range of motion (as the participant is able to perform)	Initial load: 70% 1RM<br><br>3 sets x 12 reps<br><br>2-3 minutes of rest between sets<br>1 second rest between repetitions<br><br>Time under tension/reps: 4 seconds<br>Eccentric phase: 2 seconds<br>Concentric phase: 2 seconds<br><br>Regression: decrease load (60% 1 RM) or the number of repetitions (up to 8) or range of motion (depending on the participant's ability to perform)	Initial load: 40 - 60% 1RM<br><br>3 sets x 6 reps<br><br>2-3 minutes of rest between sets<br><br>Time under tension/reps: <2 seconds<br>Eccentric phase: <1 second<br>Concentric phase: 1 second<br><br>Regression: decrease load (up to 30% 1RM)

Starting position Execution<br>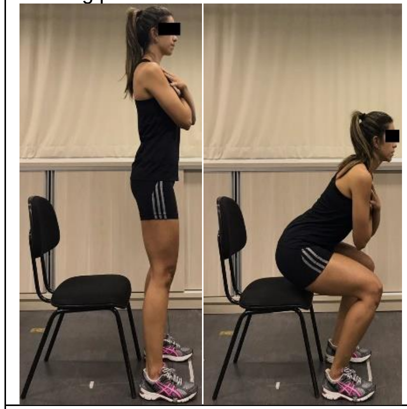	Progression: increase load (2-10% 1 RM) or the number of repetitions (up to 25) or range of motion	Progression: increase load (2-10%) or range of motion	Progression: increase the speed of the concentric phase
**Wall Sit**<br>Description: Participant starts standing, with feet shoulder-width apart. The participant performs the squat, sliding down the wall until he/she achieves 30° flexion of the hips and knees (eccentric phase), maintains the position for 60 seconds (isometric phase) and then returns to the starting position (concentric phase).<br><br>Starting position Execution<br>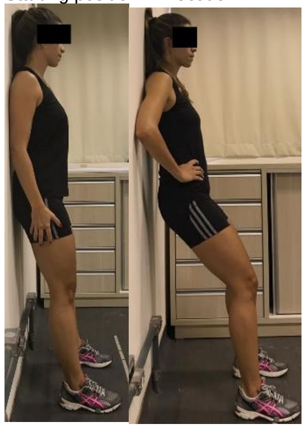	No load<br><br>3 sets x 1 reps (60 seconds)<br><br>1-minute rest between sets<br><br>Time under tension/reps: 64 seconds<br>Eccentric phase: 2 seconds<br>Isometric phase: 60 seconds<br>Concentric phase: 2 seconds<br><br>Regression: decrease knee flexion range of motion<br>Progression: increase the knee flexion range of motion		This exercise will not be performed for power gain.

**One Leg Squat**<br>Description: Participant starts in unilateral support, holding dumbbells. The participant squats around 90° of flexion of the hips and knees (eccentric phase) and returns to the starting position (concentric phase). This is performed on both sides.<br><br>Starting position Execution<br>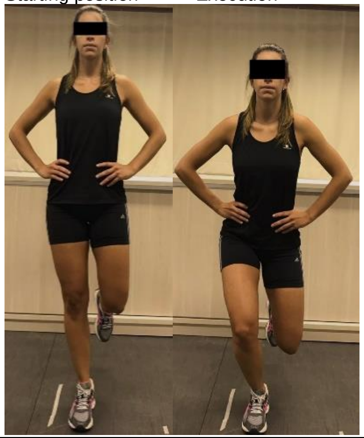	Initial load: 50% 1RM<br><br>3 sets x 20 reps<br><br>1-minute rest between sets<br>1 second rest between repetitions<br><br>Time under tension/reps: 4 seconds<br>Concentric phase: 2 seconds<br>Eccentric phase: 2 seconds<br><br>Regression: decrease load (45% 1RM) or the number of repetitions (up to 15) or range of motion (as the participant is able to perform)<br><br>Progression: increase load (2-10% 1 RM) or the number of repetitions (up to 25) or range of motion	Initial load: 70% 1RM<br><br>3 sets x 12 reps<br><br>2-3 minutes of rest between sets<br>1 second rest between repetitions<br><br>Time under tension/reps: 4 seconds<br>Concentric phase: 2 seconds<br>Eccentric phase: 2 seconds<br><br>Regression: decrease load (60% 1 RM) or the number of repetitions (up to 8) or range of motion (depending on the participant's ability to perform)<br><br>Progression: increase load (2-10%) or range of motion	Initial load: 40 - 60% 1RM<br><br>3 sets x 6 reps<br><br>2-3 minutes of rest between sets<br><br>Time under tension/reps: <2 seconds<br>Concentric Phase: <1 second<br>Eccentric Phase: 1 second<br><br>Regression: decrease load (up to 30% 1RM)<br><br>Progression: increase the speed of the concentric phase
**Lunge**<br>Description: Participant starts standing, with one foot more forward than the other, shoulder-width apart and holding dumbbells. The participant performs the squat, around 90° of flexion of the hips and knees (eccentric phase) and returns to the starting position (concentric phase).	Initial load: 50% 1RM<br><br>3 sets x 20 reps<br><br>1-minute rest between sets<br>1 second rest between repetitions<br><br>Time under tension/reps: 4 seconds<br>Eccentric phase: 2 seconds<br>Concentric phase: 2 seconds<br><br>Regression: decrease load (45% 1RM) or the number of repetitions (up to 15) or range of motion (as the participant is able to perform)	Initial load: 70% 1RM<br><br>3 sets x 12 reps<br><br>2-3 minutes of rest between sets<br>1 second rest between repetitions<br><br>Time under tension/reps: 4 seconds<br>Eccentric phase: 2 seconds<br>Concentric phase: 2 seconds<br><br>Regression: decrease load (60% 1 RM) or the number of repetitions (up to 8) or range of motion (depending on the participant's ability to perform)	Initial load: 40 - 60% 1RM<br><br>3 sets x 6 reps<br><br>2-3 minutes of rest between sets<br><br>Time under tension/reps: <2 seconds<br>Eccentric phase: <1 second<br>Concentric phase: 1 second<br><br>Regression: decrease load (up to 30% 1RM)

Starting position Execution 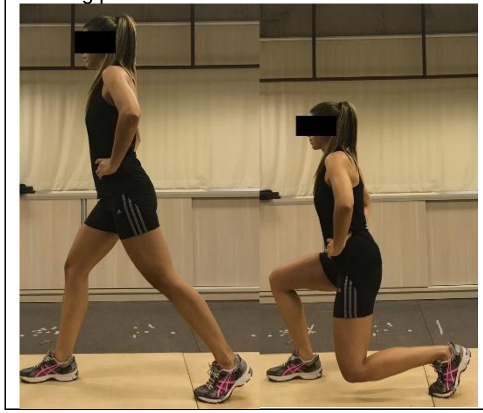	Progression: increase load (2-10% 1 RM) or the number of repetitions (up to 25) or range of motion	Progression: increase load (2-10%) or range of motion	Progression: increase the speed of the concentric phase

Trunk

<table>
<tr><th>Exercise</th><th>Resistance Training</th><th>Strength Training</th><th>Power Training</th></tr>
<tr><td>Anterior Plank
Description: Participant starts the plank on the elbows and forearms, with the weight distributed equally between the knees and forearms. The participant must keep the spine in a neutral position and the hips and knees 0° in extension.

Execution
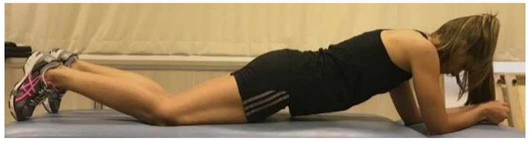
Progression
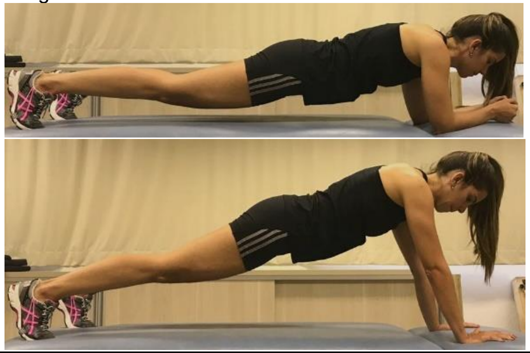</td><td colspan="2">No load

5 sets x 30 seconds

1-minute rest between sets

Time under tension/reps: 34 seconds
Concentric phase: 2 seconds
Isometric phase: 30 seconds
Eccentric phase: 2 seconds

Regression: decrease time (every 5 seconds)

Progression: increase time (every 5 seconds) or switch to foot support and then switch to hand support.</td><td>This exercise will not be performed for power gain.</td></tr>
<tr><td>Side Plank
The participant is positioned with the side to be exercised facing down toward the floor, with support on the elbow and forearm, and the knees flexed. The participant raises the hip from the ground until it reaches the neutral position (concentric phase) and then returns to the initial position (eccentric phase). This is performed on both sides.

Starting position</td><td colspan="2">No load

5 sets x 30 seconds

1-minute rest between sets

Time under tension/reps: 34 seconds
Concentric phase: 2 seconds
Isometric phase: 30 seconds
Eccentric phase: 2 seconds

Regression: decrease time (every 5 seconds)</td><td>This exercise will not be performed for power gain.</td></tr>
</table>

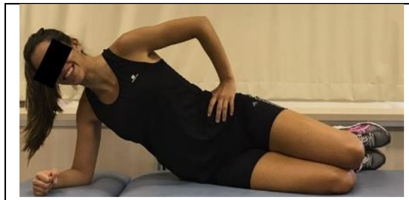<br>Execution<br>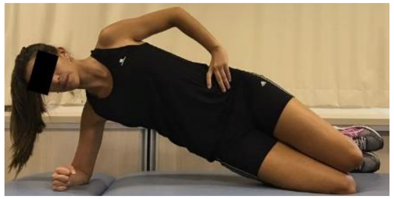<br>Progression<br>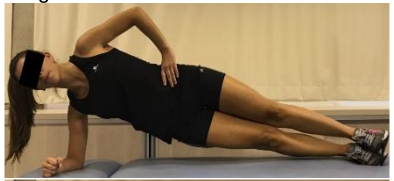<br>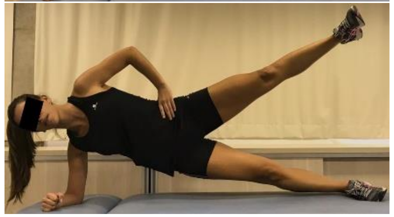	Progression: increase time (every 5 seconds) or perform with your knees extended and feet on the floor, and then abduct your hips.	
